# Multiple risk behaviour in adolescence is associated with substantial adverse health and social outcomes in early adulthood: Findings from a prospective birth cohort study

**DOI:** 10.1016/j.ypmed.2020.106157

**Published:** 2020-09

**Authors:** Rona Campbell, Caroline Wright, Matthew Hickman, Ruth R. Kipping, Michèle Smith, Theodora Pouliou, Jon Heron

**Affiliations:** a Population Health Sciences, Bristol Medical School, University of Bristol, Bristol BS8 2BN, UK; b School of Clinical Sciences, University of Bristol, Bristol BS8 2BN, UK; c College of Medicine, Swansea University, Swansea SA2 8PP, UK

**Keywords:** Adolescence, ALSPAC, Multiple risk behaviours, Adverse health outcomes, Cohort study

## Abstract

Adolescents' engage in new behaviours such as substance use and change others, such as reducing physical activity. Risks to health from these tend to be considered separately. We examined the association between multiple risk behaviours at age 16 years and outcomes in early adulthood.

5591 young people enrolled in the Avon Longitudinal Study of Parents and Children provided data on at least one of seven adverse outcomes at age ~18 years. We used logistic regression to examine associations between total number of risk behaviours and rates of depression, anxiety, problem gambling, getting into trouble with the police, harmful drinking, obesity and not in education, employment or training (NEET) at age 18 years.

We found strong associations between multiple risk behaviours and all seven adverse outcomes. For each additional risk behaviour engaged in the odds of harmful drinking increased by OR = 1.58[95%CI:1.48,1.69], getting into trouble with the police OR = 1.49[95%CI:1.42,1.57], having depression OR = 1.24[95%CI:1.17,1.31], problem gambling OR = 1.20[95%CI:1.13,1.27], NEET OR = 1.19[95%CI:1.11,1.29], anxiety OR = 1.18[95%CI:1.12,1.24] and obesity OR = 1.09[95%CI:1.03,1.15]. Neither adjustment for sex, parental socio-economic position and maternal risk behaviours, nor confining analyses to adolescents with no previous presentation of these adverse outcomes, resulted in any notable reductions in the odds ratios.

Investment in interventions and environments that effectively prevent multiple risk behaviour is likely to improve a range of health outcomes in young adults.

## Highlights

• MRB in adolescence was associated with adverse health outcomes in early adulthood.
• For each additional risk behaviour odds of an adverse outcome ranged from 1.09–1.58.
• Adjustments for potential confounders did not alter the odds of an adverse outcome.
• Association between MRB and adverse outcomes was not due to reverse causality.

## 1 Introduction

Globally, smoking tobacco, alcohol use, being physically inactive and eating an unhealthy diet are the principal causes of non-communicable diseases (NCDs)^1^ (Alwan, 2011). Interpersonal violence, substance misuse and unsafe sex are also threats to adolescent health worldwide. Injury, predominantly related to transport and vehicle-rated behaviour, is the dominant cause of death among adolescents (Mokdad et al., 2016; Viner et al., 2011). These behaviours are all modifiable through policy, environmental, community, family and individually targeted interventions, there is considerable scope to improve young people's health in both the short- and long-term.

Adolescence is a pivotal life stage characterised by physical, cognitive, social and emotional change as young people acquire the resources and capabilities needed for adult life. It is second only to infancy as a dynamic period for brain development (Andersen and Teicher, 2008). Adolescence is a time to embed healthy habits and foster resilience but engagement in behaviours such as smoking tobacco, drinking alcohol, and physical inactivity generally begin in adolescence and affect health in later life as important contributors to the epidemic of NCD (Gore et al., 2011). That many NCDs have their origin in early life is well understood by international bodies and in 2019 UNICEF issued guidance on its approach to early life prevention of NCDs which included action to influence government policy to ‘support the development of programmes for protective environments that discourage risk behaviours’ (unicef, 2019).

The approach to tackling this has been to have separate prevention programmes for separate risk behaviours. However, evidence increasingly shows that these behaviours co-exist at the population level (Buck and Frosini, 2012) and aggregate within individuals (Jackson et al., 2012). In the UK this has led to calls for public health practice to target multiple, not single risk behaviours (Buck and Frosini, 2012). National policy now encourages this (Department of Health, 2010) and there is some evidence that this is being implemented by Local Authorities for adults (Evans and Buck, 2018). However, there has been less attention given to the more proximal impact of these health risk behaviours in adolescence and on what the cumulative impact of engagement in multiple risk behaviours (MRBs) might be in the short term.

There is some emerging evidence that engagement in multiple risk behaviour may be associated with adverse health and social outcomes in early adulthood. Research in the US has shown that adolescents with higher risk profiles are more likely to be arrested, unemployed or out of school and report worse physical health (Hair et al., 2009). Two studies employing latent class analysis to investigate clustering of multiple risk behaviours in relation to mental ill health and obesity and overweight in young adulthood have shown that in Australia the class with high probabilities of smoking and binge drinking and low fruit/vegetable consumption had a substantially increased risk of psychological distress, anxiety and depression (Champion et al., 2018). And in the US all classes had greater odds of being overweight/obese compared to the ‘health conscious’ class (Laxer et al., 2017). Evidence that MRBs are associated with poorer educational outcomes is also building. An analysis of AddHealth data in the US found those with at least a college degree were less likely to belong to the cluster distinguished by their unhealthy behaviours, even after adjustments (Skalamera and Hummer, 2015). In a related study we have shown performance in General Certificate of Secondary Education (GCSE) examinations in the UK at age 16 to be equivalent to a reduction in more than one grade for every MRB engaged in (Wright et al., 2018).

We sought to explore associations between engagement in MRBs in mid-adolescence and adverse health and social outcomes in early adulthood and to our knowledge this is the first UK study to do so. When considering population health in adolescence and young adulthood a challenge is to identify age appropriate outcome measures, important in the short term, as well as measures that are known precursors of premature mortality and NCD relevant to health in the longer term. We aimed to measure the association between number of MRBs engaged in at age 16 and a series of outcome measures which met these criteria: obesity (Bitzur et al., 2016; Brown et al., 2018; Connor, 2016; Praud et al., 2016; WCRF, 2018), harmful drinking (Mokdad et al., 2016), problem gambling (Karlsson and Håkansson, 2018), getting into trouble with the police (Elonheimo et al., 2017), NEET (PHE, 2014) and depression and anxiety (Mokdad et al., 2016).

## 2 Methods

### 2.1 Study participants

Data were drawn from the Avon Longitudinal Study of Parents and Children (ALSPAC) (Boyd et al., 2013; Fraser et al., 2013), an ongoing population-based study investigating the effects of a range of influences on the health and development of children and their parents. Pregnant women resident in Avon, UK with expected dates of delivery 1st April 1991 to 31st December 1992 were invited to take part in the study. The initial number of pregnancies enrolled is 14,541 (for these at least one questionnaire has been returned or a “Children in Focus” clinic had been attended by 19/07/99). Of these initial pregnancies, there was a total of 14,676 foetuses, resulting in 14,062 live births and 13,988 children who were alive at 1 year of age.

### 2.2 Exposure – number of risk behaviours engaged in during mid-adolescence

Measures of participation in thirteen risk behaviours were derived from participants' responses to: (i) a self-completed questionnaire issued at a clinic attended at age 15 (median age 15 years and 5 months) and, (ii) a postal questionnaire administered at age 16 (median age 16 years and 7 months). These behaviours, which for brevity hereafter we refer to as occurring at age 16, represent domains of social and health risk; including: sexual health, substance use, self-harm, vehicle-related injury risk, criminal and antisocial behaviour (ASB) and physical inactivity. Each exposure variable is described in Supplementary Table S1 and earlier publications (Kipping et al., 2015; Wright et al., 2018). These 13 variables were dichotomous, a score of 1 indicating engagement in a recognised health risk behaviour. Scores were summated into a total score for each participant. We used a total multiple risk behaviour score as we have found only weak evidence of these MRBs clustering and no explanatory advantage of using latent classes over summing the risk behaviours (Wright et al., 2020).

### 2.3 Outcomes

Binary outcome indicators were derived for each of the seven outcomes of interest (Supplementary Table S1). Indicators of harmful drinking, getting into trouble with the police, having depression, problem gambling, NEET, anxiety and obesity were based on either measurements made by staff or responses to questionnaires by the young person when they attended a clinic at 17 (median age 17 years and 10 months). NEET status was derived from responses to a postal questionnaire at age 18 (median 18 years and 8 months). For brevity hereafter we refer to outcomes at age 18.

### 2.4 Potential confounders

Sex and socioeconomic status (SES) measures - maternal educational attainment, household equivalized income and parental social class - were adjusted for given the known associations between individual MRBs and both sex and SES (Kipping et al., 2015). Other potential confounders include parents' risk behaviour and similar adverse outcomes experienced by parents earlier in the children's lives. Relevant data were only available for mothers so adjustments were made for pre-pregnancy maternal obesity, postnatal maternal depression when child was 21 months, maternal cannabis use when child was age 9, and maternal smoking, harmful maternal alcohol consumption, and whether the mother had ever been in trouble with the law when the child was age 12 years.

#### 2.4.1 Statistical method

To address the possibility that adverse outcomes co-occur, and that any relationship between MRB and adverse outcomes was confined to a small group of individuals, tetrachoric correlation was used to determine the degree of correlation between all seven outcome measures at age 18 (Table 2).

In the main analysis a logistic univariable regression model was estimated for each outcome in turn with total MRB score at age ~16. Estimates were then cumulatively adjusted for the potentially confounding effects of: (i) sex; (ii) three SES measures; and (iii) relevant adverse maternal outcomes and risk behaviours earlier in the child's life.

#### 2.4.2 Exclusions

As some associations could have arisen through reverse causality, models were re-estimated after excluding participants with presentations of the outcome at an earlier stage in their life (Table 3).

Some risk behaviours at age 16 are linked to the outcomes at age 18 (e.g. hazardous alcohol consumption at 16 and harmful alcohol consumption at 18). To assess the potential impact of this on the association between exposure and outcome, the main analysis was repeated, but, for each outcome in turn, each risk behaviour was removed from the total MRB score.

The complete case samples for each binary outcome were utilised to compare models treating the exposure as categorical with more parsimonious models which measure dose response assuming a linear increase per category of the exposure tested using likelihood ratio tests. A linear trend between the number of behaviours category and the outcome was upheld in all regression models apart from that for harmful alcohol use at age 18. In the latter case, there was some indication of a J-shaped association between harmful alcohol use and exposure to MRB. We investigated a model allowing for a linear increase in the log-odds of having the outcome per additional behaviour engaged in, by taking the midpoints of the exposure category. The non-linear nature of the association between harmful alcohol consumption and the exposure was explored by adding quadratic terms in the exposure. There was no evidence that this had greater predictive power than a linear effect of the exposure on harmful alcohol consumption (likelihood ratio test yielded *p* = 0.08) so the relationship has been assumed linear hereon. Tests were used for the imputed data and similar linear trends for the numbers of MRBs and each outcome were shown.

### 2.5 Multiple imputation and exclusions

For each outcome, analyses were initially carried out using complete case samples. This produced samples that varied from 1693 (NEET) to 2249 (obesity). Multiple imputation was employed as a sensitivity analysis to examine the potential impact of non-response bias, increasing the sample to the 5591 who provided any outcome information when aged 18 (details about deriving the sample can be found in supplementary material Fig. S1 along with a more detailed discussion about missing data). Multivariate Imputation by Chained Equations (White et al., 2011) was carried out using the *ice* routine (Strack and Deutsch, 2004) in Stata version 11.2 (StataCorp., 2009) which assumes data are ‘Missing At Random’. Auxiliary variables were included in the imputation of variables with missing values. These included indicators of early life family adversity and prior measures of MRB which were more proximal to the outcomes of interest. We compared the results obtained when imputing 100 datasets and 20 cycles of regression switching and looked for indicators that all measures imputed converged to a tolerable precision. To address the possibility of reverse causality a separate analysis was performed to exclude those with presentations of the outcomes in early adolescence. Having highlighted participants with evidence of pre-existing adverse outcomes we incorporated this information as a series of moderator variables both as part of the imputation model and the analysis that followed. We were thus able to test the associations between MRB and particular outcomes, for both cases with/without those pre-existing adverse outcomes, by building interaction terms into the imputation routine. Further, by not excluding cases, we were able to maintain the same sample size, irrespective of the flagged sample which would be expected to vary in magnitude owing to missing data in these measures. A separate imputation model was run for each of the outcome measures. For simplicity we nevertheless refer to these as exclusions.

Estimates from the complete case sample and imputation sample did not differ substantially. Results reported below are for the imputation sample (for complete case see Supplementary Table S2).

## 3 Results

### 3.1 Prevalence and clustering of outcomes

Getting into trouble with the police, with a prevalence of 14.8%, was the most common adverse outcome at age 18 followed by obesity with a prevalence of 10.4% (Supplementary Table S3). Harmful drinking had the lowest prevalence at 5.6% while NEET had a prevalence of 7%. The prevalence of depression and problem gambling was similar at just over 8% and the prevalence of anxiety was somewhat higher at 11.3%. Excluding young people who had experienced these adverse outcomes earlier in their lives made minimal difference except with obesity where the prevalence reduced by a third to 6.5%.

The seven outcomes were not highly correlated with each other (Table 1). There was a moderately strong pairwise correlation between getting into trouble with the police and harmful alcohol consumption (*r* = 0.484) and a strong correlation between anxiety and depression (*r* = 0.716) but pairwise correlations between all other outcomes were weak.

**Table 1:** Tetrachoric correlation matrix of outcomes at age 18 years (pairwise deletion).

	Harmful alcohol use	Obesity	NEET	Anxiety	Depression	Trouble with police	Problem gambling
Harmful alcohol use	1						
Obesity	0.005	1					
NEET	0.183	0.040	1				
Anxiety	0.216	0.065	0.061	1			
Depression	0.203	0.135	0.111	0.716	1		
Trouble with police	0.484	0.046	0.124	0.006	0.056	1	
Problem gambling	0.183	0.107	0.141	0.005	0.001	0.280	1

### 3.2 Social patterning of outcomes

There were differences in some outcomes according to sex (see Table 2). Compared to males, females had half the odds of getting into trouble with the police (OR = 0.45[95%CI:0.38,0.54]) or problem gambling (OR = 0.54[95%CI:0.43,0.67]) but had more than twice the odds of suffering from anxiety (OR = 2.07[95%CI:1.69,2.53]) or depression (OR = 2.19[95%CI:1.70,2.81]). There were no sex differences in the prevalence of NEET, obesity or harmful drinking.

**Table 2:** Social patterning of adverse outcomes according to sex, household income, maternal educational attainment and parental social class – (imputed sample).

	Harmful drinking	Obesity	NEET	Anxiety	Depression	Trouble with police	Problem gambling
	Odds ratio [95% CI]	Odds ratio [95% CI]	Odds ratio [95% CI]	Odds ratio [95% CI]	Odds ratio [95% CI]	Odds ratio [95% CI]	Odds ratio [95% CI]
Sex							
Male (ref)							
Female	0.97 [0.74, 1.27]	1.10 [0.91, 1.33]	0.83 [0.64, 1.08]	2.07 [1.69, 2.53]	2.19 [1.70, 2.81]	0.45 [0.38, 0.54]	0.54 [0.43, 0.67]

Household income							
High (ref)							
Middle high	1.15 [0.76, 1.74]	1.33 [0.98, 1.81]	0.93 [0.60, 1.45]	1.06 [0.79, 1.42]	1.10 [0.78, 1.55]	1.08 [0.82, 1.42]	0.98 [0.71, 1.36]
Middle	1.28 [0.84, 1.96]	1.45 [1.06, 1.99]	1.60 [1.07, 2.38]	1.07 [0.79, 1.44]	1.34 [0.95, 1.89]	1.34 [1.02, 1.75]	0.95 [0.67, 1.35]
Middle low	1.14 [0.71, 1.81]	1.69 [1.24, 2.31]	1.62 [1.06, 2.47]	1.21 [0.90, 1.63]	1.07 [0.74, 1.54]	1.62 [1.23, 2.13]	1.08 [0.76, 1.52]
Low	1.45 [0.91, 2.31]	2.06 [1.51, 2.80]	2.41 [1.56, 3.71]	1.45 [1.06, 1.99]	1.54 [1.06, 2.24]	1.54 [1.12, 2.14]	0.98 [0.66, 1.47]

Maternal educational attainment							
Degree (ref)							
A level	1.10 [0.71, 1.71]	1.64 [1.16, 2.33]	1.04 [0.69, 1.57]	1.21 [0.91, 1.62]	1.28 [0.90, 1.82]	1.33 [0.99, 1.79]	1.05 [0.75, 1.48]
O level	1.40 [0.94, 2.09]	2.36 [1.69, 3.30]	1.28 [0.86, 1.90]	1.26 [0.95, 1.67]	1.39 [1.00, 1.93]	1.80 [1.34, 2.41]	1.16 [0.85, 1.60]
<O level	1.39 [0.87, 2.20]	3.16 [2.22, 4.52]	1.59 [0.99, 2.55]	1.37 [1.00, 1.87]	1.30 [0.89, 1.89]	2.21 [1.62, 3.02]	1.26 [0.87, 1.81]

Parental social class							
Professional (ref)							
Managerial and technical	0.95 [0.65, 1.40]	1.93 [1.37, 2.70]	1.29 [0.85, 1.96]	1.17 [0.88, 1.55]	1.33 [0.95, 1.87]	1.53 [1.16, 2.02]	1.07 [0.78,1.47]
Skilled non-manual	1.14 [0.74, 1.74]	2.06 [1.43, 2.96]	1.72 [1.10, 2.70]	1.33 [0.97, 1.82]	1.46 [1.00, 2.14]	1.67 [1.22, 2.29]	1.29 [0.90, 1.85]
Skilled manual, etc.	1.15 [0.70, 1.89]	2.98 [2.03, 4.38]	2.41 [1.44, 4.04]	1.47 [1.03, 2.11]	1.61 [1.05, 2.45]	1.88 [1.32, 2.69]	1.41 [0.95, 2.09]

The prevalence of harmful drinking, anxiety, depression and problem gambling did not vary according to the three measures of SES (Table 2). By contrast, getting in trouble with the police was strongly socially patterned with prevalence increasing with reduced income, low maternal educational attainment and lower social class. NEET was similarly patterned according to household income and parental social class but there was no evidence of differentiation by mother's educational attainment and the associations were the same for obesity.

### 3.3 Associations between multiple risk behaviours and health and social outcomes

Strong associations were observed between MRBs and all seven outcomes (Table 3). The strongest associations were for harmful drinking and getting into trouble with the police. In the unadjusted model the odds ratios were 1.58[95%CI:1.48,1.69] and OR = 1.49[95%CI:1.42,1.57], respectively. There was evidence of a strong association between MRBs and indicators of poor mental health. For each additional MRB, odds of having depression were OR = 1.24[95%CI:1.17,1.31], anxiety OR = 1.18[95%CI:1.12,1.24], and odds of NEET OR = 1.19[95%CI:1.11–1.29]. The weakest association was observed for obesity but even here, for each supplemental MRB, the odds of being obese at age 18 increased by 9% (OR = 1.09[95%CI:1.03,1.15]). Adjustments made for sex, parental SES, previous adverse maternal outcomes and risk behaviours did little to alter the odds of these outcomes at age 18. Similarly, excluding young people who had previously reported having some of these outcomes from the analyses (lower part of Table 3) did not alter the magnitude of increased risk associated with increasing MRB. Likewise, as shown in Table 4 removing from the total MRB score those data relating to behaviours most closely associated with each outcome had minimal impact on the magnitude of the increased risk of each outcome. For example, for harmful alcohol OR = 1.53 when no MRBs are removed from the exposure measure and OR = 1.51 when hazardous alcohol use is removed.

**Table 3:** Association between the number of risk behaviours (linear) at age 16 years and adverse health outcomes at age 18 for the imputed sample (*n* = 5591).

	Unadjusted analysis	Adjusted for sex	Adjusted for sex, and parental socio-economic status (i.e. maternal education, parental social class and household equivalized income)	Adjusted for sex, parental socio-economic status and previous adverse maternal outcomes and health risk behaviours (full details below)^a^
	Odds ratio [95% CI]	Odds ratio [95% CI]	Odds ratio [95% CI]	Odds ratio [95% CI]
Pre-exclusion				
Harmful alcohol use	1.58 [1.48, 1.69]	1.58 [1.47, 1.69]	1.59 [1.48, 1.70]	1.59 [1.48, 1.71]
Obesity	1.09 [1.03, 1.15]	1.09 [1.03, 1.15]	1.06 [1.01, 1.12]	1.08 [1.01, 1.14]
NEET	1.19 [1.11, 1.29]	1.20 [1.11, 1.29]	1.18 [1.10, 1.27]	1.17 [1.08, 1.27]
Anxiety	1.18 [1.12, 1.24]	1.18 [1.12, 1.24]	1.18 [1.12, 1.24]	1.18 [1.11, 1.24]
Depression	1.24 [1.17, 1.31]	1.24 [1.17, 1.31]	1.23 [1.17, 1.30]	1.24 [1.16, 1.31]
Trouble with police	1.49 [1.42, 1.57]	1.54 [1.46, 1.63]	1.53 [1.45, 1.62]	1.54 [1.45, 1.63]
Problem gambling	1.20 [1.13, 1.27]	1.20 [1.13, 1.28]	1.20 [1.13, 1.27]	1.21 [1.14, 1.29]

Post-exclusion^b^				
Harmful alcohol use	1.54 [1.43, 1.65]	1.54 [1.43, 1.66]	1.54 [1.43, 1.67]	1.55 [1.43, 1.68]
Obesity	1.10 [1.03, 1.18]	1.10 [1.03, 1.18]	1.08 [1.01, 1.16]	1.09 [1.02, 1.17]
Anxiety	1.17 [1.11, 1.24]	1.17 [1.11, 1.24]	1.17 [1.11, 1.23]	1.16 [1.09, 1.23]
Depression	1.23 [1.16, 1.30]	1.23 [1.16, 1.31]	1.23 [1.16, 1.30]	1.23 [1.15, 1.31]
Trouble with police	1.48 [1.40, 1.57]	1.52 [1.43, 1.62]	1.52 [1.43, 1.62]	1.52 [1.43, 1.62]

a Postnatal maternal depression when child was 8 months, pre-pregnancy maternal obesity, maternal smoking when child was age 12, maternal harmful alcohol consumption when child was age 12, maternal cannabis use when child was age 9 and maternal ever trouble with the law when child was age 12.

b Individuals with a history of harmful alcohol use (1.2%), obesity (5.4%), anxiety/depression (4.6%), and being in trouble with police (5.2%) were excluded.

**Table 4:** Odd ratios for each outcome with each risk behaviour, in turn, removed from the total multiple risk behaviour score (imputed sample).

Risk behaviour removed	Harmful alcohol	Obesity	NEET	Anxiety	Depression	Trouble with police	Gambling
	OR	OR	OR	OR	OR	OR	OR
None	1.534	1.091	1.181	1.233	1.306	1.485	1.215
Car passenger risk	1.553	1.109	1.175	1.253	1.344	1.551	1.233
Scooter risk	1.586	1.102	1.197	1.244	1.346	1.479	1.204
Cycle helmet use	1.560	1.117	1.183	1.259	1.341	1.432	1.216
Illicit drug/solvent use	1.573	1.106	1.191	1.239	1.313	1.522	1.235
Cannabis use	1.597	1.112	1.174	1.261	1.342	1.526	1.253
Criminal/antisocial behaviour	1.589	1.113	1.198	1.280	1.359	1.496	1.209
Regular tobacco smoking	1.599	1.104	1.192	1.257	1.329	1.531	1.251
Hazardous alcohol use	1.514	1.112	1.212	1.262	1.347	1.537	1.236
Sex before age 16 and/or unprotected sex^a^	1.606	1.090	1.185	1.261	1.328	1.532	1.215
Self-harm	1.561	1.068	1.204	1.192	1.271	1.555	1.246
Physical inactivity	1.554	1.063	1.191	1.228	1.295	1.537	1.221
TV viewing	1.561	1.063	1.184	1.236	1.296	1.509	1.217

a For the purposes of this analysis these two variables have been combined.

## 4 Discussion

This study of 13 risk behaviours revealed a strong association between the total MRB score at age 16 and deleterious health and social outcomes at age 18. This remained after adjustment for confounders and was unaltered following exclusion of participants with evidence of pre-existing adverse outcomes and if the MRB most closely associated with the outcome was dropped. The strength of this association is such that the odds of a young person engaged in one risk behaviour age 16 suffering from depression by age 18 was 1.24 and with a change to two risk behaviours (the norm) the odds increase to 1.53 (x^2^) and for four risk behaviours to 2.36 (x^4^). The comparable odds for getting into trouble with the police are 1.49, 2.22 and 4.49, respectively. The weak correlation between outcomes measures excludes the possibility that the association is confined to a small group experiencing multiple adversity.

### 4.1 Limitations

Firstly, most of our data relied on self-report and are therefore potentially subject to social desirability bias. However, by adolescence ALSPAC participants' have probably developed trust in the maintenance of their anonymity reducing such bias. Secondly, while including a wide range of risk behaviours we were unable to include data on eating behaviour as these were not collected at age 16. Thirdly, we were only able to adjust for the potentially confounding effect of mother's risk behaviour and related morbidity, not father's. However, evidence from the TRAILS study suggests that mother's risk behaviour and emotional support may be more important in influencing children's risk behaviour than father's (de Winter et al., 2016). Fourthly, MRB was represented by summation of 13 dichotomous risk behaviour variables, an approach highlighted as problematic (McAloney et al., 2013) because we lack agreed standards for distinguishing between a level of engagement that constitutes risky behaviour from that signifying no risk. However, we dichotomised our risk variables with reference to agreed health guidelines wherever possible (Kipping et al., 2015). MRB number may also be an over-simplification of exposure, but there was no strong evidence for a more refined profile from our latent class analyses in ALSPAC (Wright et al., 2020). Finally, there was substantial missing data, reducing power and potentially introducing bias. Our imputation sample represents those participants who provided data on any outcome measure. However, for this type of bias to be problematic, our outcome measures would have to be conditionally related to whether a participant remains in the sample, or missing not at random (MNAR) (Hughes et al., 2019; Sterne et al., 2009). We assume that our data is missing at random (MAR), which we discuss in more detail in the supplementary material.

### 4.2 Implications

We must take greater account of evidence suggesting adolescents engaging in more MRBs, are associated with increased odds of poor health and social outcomes, even in early adulthood. Interventions need to be developed and deployed to address this. Further research is required into the antecedents of MRBs, including the role of adverse childhood experiences (Felitti et al., 1998), to understand which factors are associated with increased engagement in MRBs and which appear protective. Opportunities for earlier intervention, before engagement in MRBs begins, can thus be identified. Research is also required into whether the associations observed in ALSPAC are evident in similar cohort studies and if they persist. Systematic reviews suggest effective interventions are available for children and young people, especially in the school context. For example, a Cochrane review showed that the WHO health promoting schools framework ameliorates aspects of student health, that would be important at the population level (Langford et al., 2015). Schools can provide a useful context for preventing MRBs, not only because they are practical, but also because of the importance of, and interplay between peer influence and school context in shaping adolescent MRBs (Hale et al., 2014). Evidence shows that interventions targeting multiple-substance use can also be effective for other MRBs and would provide an excellent basis for MRB prevention programmes. A Cochrane Review of individual, family, and school-level interventions targeting adolescent MRBs found that universal school-based interventions are most effective in preventing tobacco use, alcohol consumption, illicit drug use and antisocial behaviour, and increasing physical activity among young people, but did not find strong evidence of benefit for family or individual-level interventions for the MRB studied (MacArthur et al., 2018). The evidence for social patterning in the way adolescent MRBs cluster suggests structural level interventions are required (Meader et al., 2016). Therefore, in addition to targeted interventions, there should be ‘broader social change (to address the impact of pricing and availability of substances, marketing, media, culture and social norms on risk behaviour) and efforts to reduce marginalisation, social exclusion and the vulnerability of young people during periods of transition.’ (Jackson et al., 2010). However, more evidence is required to evaluate the effects of community- and population-level interventions, including media interventions, policies, laws and regulations.

### 4.3 Conclusion

Globally, declines in mortality for young people in the last half-century have been less than those for children aged under 5 years (Viner et al., 2011) and action to improve the health and wellbeing of this age group is overdue. We have shown that adolescent multiple risk behaviour is strongly associated with various adverse health and social outcomes at age 18 years. Investment in interventions and environments that effectively prevent multiple risk behaviours would improve health and social outcomes in adulthood.

## Ethics approval and consent to participate

Ethical approval for the study was obtained from the ALSPAC Ethics and Law Committee and the Local Research Ethics Committees.

## Consent for publication

Informed consent for the use of data collected via questionnaires and clinics was obtained from participants following the recommendations of the ALSPAC Ethics and Law Committee at the time.

## Availability of data and material

Please note that the study website contains details of all the data that is available through a fully searchable data dictionary and variable search tool and reference the following webpage: http://www.bristol.ac.uk/alspac/researchers/our-data/.

## Funding

This work was supported by The Centre for the Development and Evaluation of Complex Interventions for Public Health Improvement (DECIPHer), a UKCRC Public Health Research Centre of Excellence. Joint funding (MR/KO232331/1) from the 10.13039/501100000274British Heart Foundation; 10.13039/501100000289Cancer Research UK; 10.13039/501100000269Economic and Social Research Council; 10.13039/501100000265Medical Research Council; the Welsh Government; and the 10.13039/100010269Wellcome Trust, under the auspices of the UK Clinical Research Collaboration, is gratefully acknowledged. CW is funded by a Cancer Research UK Population Research Postdoctoral Fellowship (C60153/A23895). The UK Medical Research Council and Wellcome (Grant ref.: 102215/2/13/2) and the University of Bristol provide core support for ALSPAC.

## Authors' contributions

All authors, except CW devised the design of the study, all authors discussed the analysis. JH, TP & MS undertook the analyses. RC wrote the first draft of the manuscript. RK, JH, CW and MH edited the paper. CW revised the manuscript.

## Declaration of competing interest

Not applicable.

## Appendix A Supplementary data

Supplementary material Image 1

## Footnotes

1 Non-communicable diseases (NCDs) are diseases that cannot be transmitted from one sufferer to another. They tend to be chronic and result from a combination of genetic, physiological, environmental and behavioural factors. Common NCDs include cardiovascular diseases, cancers, chronic respiratory diseases (such as chronic obstructive pulmonary disease and asthma) and diabetes (WHO, 2018).
