# Supplementary material for: Multiple risk behaviour in adolescence is associated with substantial adverse health and social outcomes in early adulthood: Findings from a prospective birth cohort study
Source: Prev Med. 2020 Sep;138:106157. doi: 10.1016/j.ypmed.2020.106157 (PMC7378566; doi:10.1016/j.ypmed.2020.106157)
Supplement: Supplementary file 1 — Supplementary material [file mmc1.docx]

# Supplementary material: deriving the sample

## As the ALSPAC participants approached late adolescence, 10,482 individuals and their families were still considered to be actively participating in the study and continued to be asked to contribute either to questionnaires or by attending research clinics. The remaining 3,316 had either moved home and not provided a forwarding address, explicitly asked ALSPAC that they wished to cease with participation or had failed to provide any data for a number of collection waves.

## ALSPAC collects a wealth of data, and has done so for many years, therefore participants are encouraged to contribute where they can and not feel burdened to return every questionnaire. It is also possible to opt out of one or both types of data collection (questionnaire or clinic) hence the slightly different samples involved in these two collection methods.

## With reference to Supplementary Figure S1, of the starting sample 13,798 participants (enrolled cohort, singletons and first-born twins alive at 1 year) 9,443 participants (68.4%) were invited to attend a clinic and 9,380 (68.0%) were asked to complete a postal questionnaire at age ~18 years. Of these participants 4,830 (51.1%) attended the clinic and 3,200 (34.1%) completed the questionnaire. Of these participants 5,591 provided information on at least one outcome and form the basis of the imputation sample. 2,446 (43.7%) participants additionally provided data on the exposure measure (MRBs at age ~16 years). Complete case samples range from 2,249 for the outcome obesity (highest complete case sample) to 1,693 for the outcome NEET (lowest complete case sample).

There are several reasons we chose the imputation sample we did. While attrition has resulted in a substantial reduction of the sample size, this should not influence our decision regarding what an appropriate imputation sample is. We are not able, for example, to pick an earlier point in the data collection process, simply because it will bolster the sample size, if it means compromising the imputation model, rather we must base it on the availability of the exposure and outcome measures. As demonstrated in a recent paper, the proportion of missing data provides limited information about the bias and efficiency gains that can be made using multiple imputation and therefore should not guide decisions on multiple imputation (Madley-Dowd et al., 2019). Further, it was the first time that gambling and NEET were collected in ALSPAC. With that mind, we felt it was more appropriate to impute the exposure measure (MRBs at age 16 years) which we have multiple auxiliary variables for, than to try imputing measures for which we have no additional information.

# Supplementary material: missing data

The implications of missing data and any associated bias depend on the nature of the missingness. We assume that our data are missing at random (MAR) i.e. that any systematic differences between missing and observed values can be explained by differences in observed data (Sterne et al., 2009). Specifically, we undertook a detailed analysis of covariates associated with missingness and observe that we have fewer observations for those from lower socio-economic groups (see Supplementary Table S6). Here, missing outcomes data are higher than observed outcomes data, but only because those from lower socioeconomic backgrounds are more likely to have missed the clinics/questionnaires at age 18 years. This does not lead to bias because we have included all variables associated with missingness in the multivariable multiple imputation (under a missing at random assumption) (Sterne et al., 2009). As discussed in Hughes et al.’s recent paper (Hughes et al., 2019), the presence of bias in the estimated parameter of interest (here the association between MRB and a range of different outcome variables) hinges on whether, in the context of this model, the outcome is related to missingness. Phrased another way, when conditioning on the independent variables in the regression model (MRB, measures of SES, prior sub-clinical symptoms predictive of the outcome, etc.) there should not be a systematic difference in the outcome measure between those participants included in the model and those excluded. This is more subtle and nuanced than stating that respondents and non-respondents are different so bias must be present. The assumption we are making here is that any differences in the outcome measures can be adequately explained by the other variables included.

**Supplementary Table S1: Derivation and categorisation of exposure, outcome and potential confounding variables and thresholds for exclusion for participants who had already had presentations of an outcome at an earlier stage in their life**

| **Variables** | **How each variable was derived and categorised** |
| --- | --- |
| **Exposure variables** | *These 13 variables each had dichotomous categories with a score of 1 indicating engagement in a recognised health risk behaviour. Scores for these variables were summated into total score for each participant which could have a value of between 0 and 13* |
| Physical inactivity | Young person had typically, over the past year, exercised less than 5 times per week. |
| TV viewing | Young person spent 3 or more hours watching television on average per day across the week. |
| Car passenger risk | Young person had been a car passenger at least once in their lifetime where the driver (a) had consumed alcohol or (b) did not have a valid licence or (c) the young person chose not to wear a seat belt last time they travelled in a car, van or taxi. |
| Cycle helmet use | Young person reported that they had last ridden a bicycle in the preceding four weeks and that they had failed to wear a helmet on the most recent occasion. |
| Scooter risk | Young person had driven a motorbike/ scooter off road or without a licence on a public road at least once. |
| Criminal/Antisocial behaviour | Young person reported that at least once in the past year they had undertaken at least one of the following 7 offences:-carried a weapon; physically hurt someone on purpose; stolen something; sold illicit substances to another person; damaged property belonging to someone else either by using graffiti, setting fire to it or destroying or damaging it in another fashion; subjected someone to verbal or physical racial abuse; or been rude/rowdy in a public place. |
| Hazardous alcohol consumption | In the past year had scored eight or more on the Alcohol Use Disorders Identification Test (AUDIT) (25) indicating hazardous alcohol consumption. |
| Regular tobacco smoking | Had ever smoked and was regularly smoking i.e. smoking at least one cigarette per week. |
| Cannabis use | Those who reported using cannabis “sometimes but less often than once a week” or more regular use were classified as occasional users. |
| Illicit drug/solvent use | In the year since their 15th birthday, young person had either been a regular user (i.e. used five or more times) one or more illicit drugs (excluding cannabis) including amphetamines, ecstasy, LSD, cocaine, ketamine or inhalants including aerosols, gas, solvents and poppers. |
| Self-harm | Young people who said they had purposely hurt themselves in some way in their lifetime. |
| Penetrative sex before age 16 | Young person reported having had penetrative sex in the preceding year and that they were under 16 at the time. |
| Unprotected sex | Penetrative sex without the use of contraception on the last occasion they had had sex in the past year. |
| **Outcome variables** |  |
| Harmful alcohol use | Adolescents completed the 10-item Alcohol Use Disorders Identification Test (AUDIT) (Saunders et al., 1993). We used a cut-off of 16 units or above to indicate harmful use. |
| Obesity | Height and weight measurements of adolescents when attending a clinic circa age 17 were used to calculate individual BMI scores. Age and sex appropriate cut points for the 95th percentile UK1990 BMI reference curves (Cole et al., 1995) were determined and used to derive the obesity indicator. |
| Not in Education, Employment or Training (NEET) | A binary measure was created on the basis of responses to questions about any form of paid work, training for a profession, apprenticeship, further study, voluntary work or similar activities the young person may have been involved in at age 18. Part or full-time engagement in any of these counted towards not being in the group of interest. |
| Depression and Anxiety | Depression and anxiety were measured using the Clinical Interview Schedule-Revised (CIS-R), a self-administered computerized interview which derives diagnoses based on ICD-10 criteria for depression and anxiety disorder (Lewis et al., 1992). The computerized version shows close agreement with the interviewer administered version (Bell et al., 2005; Patton et al., 1999). A binary variable indicating a primary diagnosis of major depression was taken as the depression outcome measure. A binary variable indicating a primary or secondary diagnosis of anxiety was taken as the anxiety outcome measure. |
| Trouble with the police | Adolescents were asked whether or not they had been in trouble with the police in the last year. Adolescents were assigned a score of 1 if their response was positive. |
| Problem gambling | The Problem Gambling Severity Index (PGSI)(Holtgraves, 2009), derived from the longer Canadian Problem Gambling Inventory (Ferris J, 2001), was administered to those who reported engagement in any of 16 types of gambling (e.g. lottery/horse racing/fruit machines) in the past year. For the current analysis, a problem gambler was defined as someone with a PGSI score of 1 or more. |
| **Potential confounding variables** |  |
| Sex | Recorded as male or female at birth. |
| Maternal educational attainment* | Mother’s self-reported highest achievement in nationally established academic qualifications by the time of their pregnancy categorised into: below O-level (examination taken at the end of compulsory secondary schooling in a range of subjects usually at aged 16 at that time); O-level or equivalent; A-level (a secondary education higher examination typically taken at age 18) or equivalent; University Degree. |
| Household equivalized income* | A household’s disposable income, categorised into quintiles. It is based on income declared when the child was aged 2 to 4 years of age and estimated additional income obtained through housing and council tax benefits to give final household disposable income. This was adjusted for family size and composition (Gregg et al., 2008; Melotti et al., 2011). |
| Parental social class* | This was originally a six class categorical variable assigned according to the Registrar General’s Social Class classification and captured the highest social class of either the mother or father based on their occupation. Due to low numbers of observations for the lowest social class categories, the six classes were regrouped into four as follows: I (professional), II (managerial and technical), IIIN (skilled non-manual), all lower remaining class categories combined i.e. III-NM, IV, V (skilled manual, partly skilled and unskilled respectively). |
| Pre-pregnancy maternal obesity | Maternal BMI of 30 or greater, collected via postal questionnaire shortly after enrolment. |
| Postnatal maternal depression when child was 21 months | Continuous score from the Edinburgh Postnatal Depression Scale. |
| Maternal cannabis use when child aged 9 | Yes or No. |
| Maternal smoking when child aged 12 | Yes or No |
| Maternal harmful alcohol consumption when child aged 12 | Evidence of bingeing and high weekly alcohol consumption derived from a detailed record of beers, wines and spirits consumed in the previous week. |
| Maternal ever trouble with the law when child aged 12 | Yes or No. |
| **Thresholds for exclusions for presentations of an outcome at an earlier stage in life^[[1]](#footnote-1)^** | |
| Obesity | Those at age 15 with a BMI on or above the 98th percentile reference curves |
| Harmful alcohol consumption | Those reporting harmful alcohol use at age 16 (AUDIT); |
| Anxiety and depression | Those who were classified as suffering from anxiety or depression according to the CIS-R evaluation scale at age 15 |
| Trouble with the police | Those who reported that they had been in trouble with the police for engaging in at least one of the following: been arrested (in trouble with the police in the last year) or convicted of a criminal offence (on trial in court, got police caution, got court fine, got community service order, received an antisocial behavior order (ASBO), been in a secure unit, been in prison, been in mediation as offender). |
| Under-age smoking, alcohol, cannabis or other illicit drug | Use on at least one of three measurement occasions in their early teens (13/14/15 years) |

**Supplementary Table S2: Association between number of risk behaviours (linear) at 16 years & adverse health outcomes at age 18. (Complete case)**

|  | Unadjusted | Adjusted for sex | Adjusted for sex, and parental socio-economic status (i.e. maternal education, parental social class and household equivalised income) | Adjusted for sex, parental socio-economic status and previous adverse maternal outcomes and health risk behaviours (full details below)^6^ |
| --- | --- | --- | --- | --- |
| Pre-exclusion | **OR [95% CI]** | **OR [95% CI]** | **OR [95% CI]** | **OR [95% CI]** |
| Harmful drinking | 1.55 [1.40, 1.71] | 1.55 [1.40, 1.71] | 1.54 [1.37, 1.72] | 1.51 [1.32, 1.73] |
| Obesity | 1.09 [1.02, 1.17] | 1.09 [1.02, 1.17] | 1.09 [1.01, 1.19] | 1.08 [0.97, 1.20] |
| NEET ^3^ | 1.17 [1.05, 1.31] | 1.17 [1.05, 1.31] | 1.19 [1.06, 1.34]  p=0.003 | 1.19 [1.03, 1.38] |
| Anxiety | 1.24 [1.16, 1.33] | 1.24 [1.16, 1.33] | 1.24 [1.15, 1.33] | 1.22 [1.12, 1.33] |
| Depression | 1.31 [1.22, 1.42] | 1.32 [1.22, 1.42] | 1.36 [1.25, 1.47] | 1.30 [1.17, 1.44] |
| Trouble with police | 1.50 [1.39, 1.60] | 1.54 [1.43, 1.66] | 1.50 [1.39, 1.63] | 1.53 [1.39, 1.69] |
| Gambling | 1.22 [1.12, 1.32] | 1.22 [1.12, 1.33] | 1.22 [1.12, 1.34] | 1.24 [1.10, 1.39] |
| Post-exclusion (Individuals with a history of harmful alcohol use (0.6%), obesity (5.5%), anxiety / depression (4.6%), trouble with police (4.7%) were excluded)^[[2]](#footnote-2)^ | | | | |
| Harmful drinking | 1.52 [1.36, 1.68] | 1.51 [1.36, 1.68] | 1.49 [1.32, 1.68] | 1.45 [1.26, 1.68] |
| Obesity | 1.08 [0.97, 1.20] | 1.08 [0.97, 1.20] | 1.10[ 0.98, 1.23] | 1.14 [0.99, 1.30] |
| Anxiety | 1.21 [1.13, 1.30] | 1.21 [1.13, 1.30] | 1.21[1.11, 1.31] | 1.21 [1.10, 1.33] |
| Depression | 1.28 [1.18, 1.38] | 1.28 [1.18, 1.39] | 1.32 [1.21, 1.44] | 1.26 [1.13, 1.41] |
| Trouble with police | 1.47 [1.36, 1.59] | 1.50 [1.38, 1.63] | 1.47 [1.35, 1.61] | 1.49 [1.34, 1.65] |

**Supplementary Table S3 Prevalence of adverse outcomes at age 18 years for complete case and imputed data samples before and after exclusion of individuals with evidence of a similar adverse outcome prior to age 18.**

|  | Numerator and dominator | Prevalence in complete case data set | Prevalence in imputed data set |
| --- | --- | --- | --- |
| *Pre exclusion* |  |  |  |
| Harmful drinking | 78/2004 | 3.9% | 5.6% |
| Obesity | 189/2249 | 8.4% | 10.4% |
| NEET | 82/1693 | 4.8% | 7.0% |
| Anxiety | 201/2136 | 9.4% | 11.3% |
| Depression | 152/2136 | 7.1% | 8.0% |
| Trouble with police | 209/1926 | 10.9% | 14.8% |
| Gambling | 136/1842 | 7.4% | 8.8% |
|  |  |  |  |
| *Post-exclusion* |  |  |  |
| Harmful drinking | 72/1991 | 3.6% | 5.2% |
| Obesity | 87/2126 | 4.1% | 6.5% |
| Anxiety | 175/2038 | 8.6% | 10.6% |
| Depression | 134/2038 | 6.6% | 7.5% |
| Trouble with police | 163/1835 | 9.8% | 13.4% |

**Supplementary Table S4 Prevalence of all risk behaviours at age ~16 years for imputed data sample.**

|  | Numerator and dominator | Prevalence in imputed data set |
| --- | --- | --- |
| Physical inactivity | 2905/3908 | 74.4 |
| Criminal/antisocial behaviour | 1834/4059 | 45.2 |
| Hazardous alcohol drinking | 1287/3800 | 33.9 |
| Car passenger risk | 1123/3899 | 28.8 |
| Cycle-helmet risk | 928/3935 | 23.6 |
| Daily TV viewing (3+ hours) | 786/3816 | 20.6 |
| Self-harm | 740/3936 | 18.8 |
| Scooter risks | 617/3754 | 16.4 |
| Sex before age 16 years | 604/3888 | 15.5 |
| Tobacco smoking (weekly) | 449/3932 | 11.4 |
| Cannabis use | 379/3931 | 9.6 |
| Drug/solvent use | 172/3744 | 4.6 |
| Unprotected sex | 55/3914 | 1.4 |

**Supplementary Table S5 Prevalence of adverse outcomes according to sex, household income, maternal educational attainment and parental social class (complete case)**

|  | Harmful drinking | | Obesity | | NEET | | Anxiety | | Depression | | Trouble with police | | Problem gambling | |
| --- | --- | --- | --- | --- | --- | --- | --- | --- | --- | --- | --- | --- | --- | --- |
|  | n (%) | Odds ratio  [95% CI] | n (%) | Odds ratio  [95% CI] | n (%) | Odds ratio  [95% CI] | n (%) | Odds ratio  [95% CI] | n (%) | Odds ratio  [95% CI] | n (%) | Odds ratio  [95% CI] | n (%) | Odds ratio  [95% CI] |
| *Sex* |  |  |  |  |  |  |  |  |  |  |  |  |  |  |
| Male (ref) | 94 (5.5%) |  | 198 (9.7%) |  | 89 (7.8%) |  | 125 (6.7%) |  | 81 (4.3%) |  | 349 (21.7%) |  | 194 (13.0%) |  |
| Female | 111 (5.1%) | 0.92 [0.70, 1.23] | 273 (10.6%) | 1.10 [0.91, 1.34] | 112 (5.5%) | 0.69 [0.52, 0.92] | 353 (14.8%) | 2.42 [1.95, 2.99] | 253 (10.6%) | 2.61 [2.02, 3.38] | 170 (8.2%) | 0.32 [0.26, 0.39] | 108 (5.3%) | 0.37 [0.29, 0.48] |
|  |  |  |  |  |  |  |  |  |  |  |  |  |  |  |
| *Household income* | |  |  |  |  |  |  |  |  |  |  |  |  |  |
| High (ref) | 39 (4.6%) |  | 71 (7.2%) |  | 36 (4.9%) |  | 96 (10.4%) |  | 62 (6.7%) |  | 94 (11.6%) |  | 69 (8.5%) |  |
| Middle high | 43 (5.4%) | 1.20 [0.77, 1.87] | 86 (9.3%) | 1.34 [0.96, 1.85] | 24 (3.6%) | 0.73 [0.43, 1.24] | 92 (10.9%) | 1.05 [0.78, 1.42] | 64 (7.6%) | 1.13 [0.79, 1.63] | 89 (11.9%) | 1.03 [0.76, 1.41] | 59 (8.3%) | 0.97 [0.68, 1.40] |
| Middle | 41 (5.6%) | 1.24 [0.79, 1.94] | 81 (9.8%) | 1.40 [1.01, 1.96] | 46 (7.8%) | 1.66 [1.06, 2.61] | 81 (10.4%) | 1.00 [0.73, 1.37] | 72 (9.2%) | 1.41 [0.99, 2.01] | 100 (14.5%) | 1.30 [0.96, 1.75] | 51 (8.0%) | 0.94 [0.65, 1.38] |
| Middle low | 28 (4.4%) | 0.97 [0.59, 1.60] | 86 (11.4%) | 1.66 [1.20, 2.31] | 33 (6.5%) | 1.36 [0.84, 2.21] | 82 (11.7%) | 1.14 [0.84, 1.56] | 44 (6.3%) | 0.93 [0.63, 1.39] | 112 (18.6%) | 1.75 [1.30, 2.36] | 55 (9.4%) | 1.13 [0.78, 1.63] |
| Low | 30 (6.5%) | 1.46 [0.89, 2.38] | 76 (13.7%) | 2.07 [1.47, 2.91] | 44 (11.6%) | 2.56 [1.62, 4.06] | 75 (14.8%) | 1.50 [1.08, 2.07] | 54 (10.7%) | 1.66 [1.13, 2.43] | 65 (15.2%) | 1.37 [0.97, 1.92] | 30 (7.3%) | 0.85 [0.54, 1.32] |
|  |  |  |  |  |  |  |  |  |  |  |  |  |  |  |
| *Educational attainment* | |  |  |  |  |  |  |  |  |  |  |  |  |  |
| Degree (ref) | 33 (4.4%) |  | 44 (5.2%) |  | 39 (5.6%) |  | 73 (9.3%) |  | 47 (6.0%) |  | 67 (9.5%) |  | 56 (7.8%) |  |
| A level | 48 (4.6%) | 1.03 [0.66, 1.63] | 106 (8.5%) | 1.68 [1.17, 2.41] | 45 (5.2%) | 0.93 [0.60, 1.45] | 131 (11.4%) | 1.26 [0.93, 1.70] | 92 (8.0%) | 1.37 [0.95, 1.97] | 120 (11.9%) | 1.29 [0.94, 1.77] | 77 (8.0%) | 1.03 [0.72, 1.48] |
| O level | 76 (5.9%) | 1.37 [0.90, 2.08] | 177 (11.8%) | 2.42 [1.72, 3.41] | 65 (6.5%) | 1.18 [0.78, 1.78] | 165 (11.7%) | 1.30 [0.97, 1.74] | 123 (8.8%) | 1.51 [1.07, 2.14] | 192 (15.8%) | 1.79 [1.33, 2.40] | 101 (8.8%) | 1.15 [0.82, 1.61] |
| <O level | 39 (5.7%) | 1.30 [0.81, 2.10] | 126 (15.0%) | 3.20 [2.23, 4.57] | 42 (7.9%) | 1.44 [0.92, 2.27] | 96 (12.5%) | 1.39 [1.01, 1.92] | 60 (7.8%) | 1.33 [0.89, 1.97] | 124 (19.3%) | 2.28 [1.66, 3.13] | 57 (9.3%) | 1.22 [0.83, 1.79] |
|  |  |  |  |  |  |  |  |  |  |  |  |  |  |  |
| *Parental social class* | |  |  |  |  |  |  |  |  |  |  |  |  |  |
| Professional (ref) | 36 (5.2%) |  | 40 (5.2%) |  | 28 (4.6%) |  | 68 (9.6%) |  | 40 (5.6%) |  | 61 (9.4%) |  | 51 (7.7%) |  |
| Managerial and tech. | 71 (4.3%) | 0.81 [0.54, 1.22] | 202 (10.4%) | 2.14 [1.51, 3.04] | 75 (5.5%) | 1.20 [0.77, 1.87] | 197 (10.8%) | 1.15 [0.86, 1.53] | 142 (7.8%) | 1.42 [0.99, 2.04] | 228 (14.5%) | 1.64 [1.21, 2.21] | 117 (7.7%) | 1.00 [0.71, 1.41] |
| Skilled non-manual | 44 (5.3%) | 1.03 [0.65, 1.61] | 102 (10.3%) | 2.11 [1.45, 3.08] | 50 (7.5%) | 1.67 [1.04, 2.68] | 110 (12.0%) | 1.28 [0.93, 1.77] | 75 (8.2%) | 1.49 [1.00, 2.21] | 115 (14.7%) | 1.67 [1.20, 2.32] | 70 (9.7%) | 1.28 [0.88, 1.87] |
| Skilled manual, etc. | 23 (5.2%) | 0.99 [0.58, 1.70] | 83 (15.1%) | 3.27 [2.21, 4.86] | 33 (9.6%) | 2.18 [1.30, 3.68] | 65 (13.1%) | 1.42 [0.99, 2.03] | 45 (9.0%) | 1.66 [1.07, 2.59] | 69 (16.4%) | 1.90 [1.31, 2.75] | 40 (9.9%) | 1.31 [0.85, 2.03] |
|  |  |  |  |  |  |  |  |  |  |  |  |  |  |  |

**Supplementary Table S6: Relationship between outcomes and SES measures and level of response**

|  | n |  | No outcome data^a^  (n=8,207) | Partially available^b^  (n=3,958) | Complete cases^c^  (n=1,633) |
| --- | --- | --- | --- | --- | --- |
| *Parental social class* |  |  |  |  |  |
| Professional | 1513 |  | 479 (8.8%) | 544 (15.2%) | 490 (20.9%) |
| Managerial & technical | 4744 |  | 2067 (38.0%) | 1568 (43.9%) | 1109 (47.3%) |
| Skilled non-manual | 2897 |  | 1487 (27.3%) | 899 (24.9%) | 521 (22.2%) |
| Skilled manual/ Part or unskilled | 2201 |  | 1405 (25.8%) | 570 (16.0%) | 226 (9.6%) |
|  |  |  |  |  |  |
| *Maternal educational attainment* | |  |  |  |  |
| Degree | 1576 |  | 478 (7.9%) | 556 (14.5%) | 542 (23.1%) |
| A level | 2760 |  | 1064 (17.5%) | 1000 (26.0%) | 696 (29.7%) |
| O-level | 4241 |  | 2117 (34.9%) | 1332 (34.7%) | 792 (33.8%) |
| < O-level | 3682 |  | 2410 (39.7%) | 956 (24.9%) | 316 (13.4%) |
|  |  |  |  |  |  |
| *Household equivalized income* | |  |  |  |  |
| Top 20% | 1994 |  | 615 (15.0%) | 712 (21.0%) | 667 (28.4%) |
| Upper middle 20% | 1962 |  | 695 (16.9%) | 677 (20.1%) | 590 (25.2%) |
| Middle 20% | 1952 |  | 799 (19.5%) | 692 (20.6%) | 461 (19.7%) |
| Lower middle 20% | 1945 |  | 903 (22.0%) | 652 (19.4%) | 390 (16.6%) |
| Lowest 20% | 1963 |  | 1092 (26.6%) | 633 (18.8%) | 238 (10.1%) |

^a^ Those cases with a measure of the SES category shown but missing all 13 MRB measures

^b^ Sub-sample of cases with at least 1 MRB outcome and at least one SES measure not missing

^c^ Cases with measurements of all 13 MRB outcomes and all 3 SES measures

## Supplementary figure S1: Deriving the sample


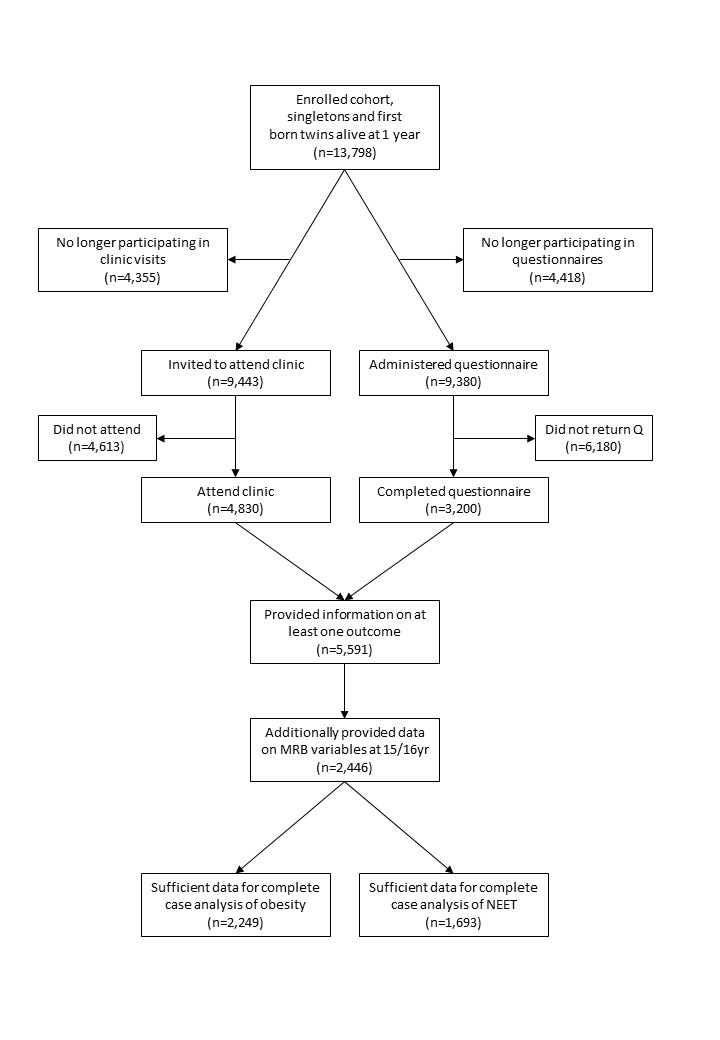


REFERENCES

Bell, T., Watson, M., Sharp, D., Lyons, I., Lewis, G., 2005. Factors associated with being a false positive on the General Health Questionnaire. Social psychiatry and psychiatric epidemiology 40:402-7.

Cole, T.J., Freeman, J.V., Preece, M.A., 1995. Body mass index reference curves for the UK, 1990. Archives of Disease in Childhood 73:25-29.

Ferris J, W.H., 2001. The Canadian Problem Gambling Index. Canadian Centre on Substance Abuse, p. 59.

Gregg, P., Propper, C., Washbrook, E., 2008. Understanding the Relationship between Parental Income and Multiple Child Outcomes: a decomposition analysis, Bristol, UK.

Holtgraves, T., 2009. Evaluating the problem gambling severity index. Journal of gambling studies/co-sponsored by the National Council on Problem Gambling and Institute for the Study of Gambling and Commercial Gaming 25:105-20.

Lewis, G., Pelosi, A.J., Araya, R., Dunn, G., 1992. Measuring psychiatric disorder in the community: a standardized assessment for use by lay interviewers. Psychological medicine 22:465-86.

Madley-Dowd, P., Hughes, R., Tilling, K., Heron, J., 2019. The proportion of missing data should not be used to guide decisions on multiple imputation. J Clin Epidemiol 110:63-73.

Melotti, R., Heron, J., Hickman, M., Macleod, J., Araya, R., Lewis, G., 2011. Adolescent alcohol and tobacco use and early socioeconomic position: The ALSPAC birth cohort. Pediatrics 127:e948-e55.

Patton, G.C., Coffey, C., Posterino, M., Carlin, J.B., Wolfe, R., Bowes, G., 1999. A computerised screening instrument for adolescent depression: population-based validation and application to a two-phase case-control study. Social psychiatry and psychiatric epidemiology 34:166-72.

Saunders, J.B., Aasland, O.G., Babor, T.F., de la Fuente, J.R., Grant, M., 1993. Development of the Alcohol Use Disorders Identification Test (AUDIT): WHO Collaborative Project on Early Detection of Persons with Harmful Alcohol Consumption--II. Addiction 88:791-804.

1. Items relating to a criminal offence such as theft/stealing were not available. Earlier data for NEET and for gambling were also not available so exclusions for these were not possible.

   * These SES measures were derived from survey responses made by the main parent/carer throughout pregnancy and the early years of life (Melotti et al., 2011). [↑](#footnote-ref-1)
2. Postnatal maternal depression when child was 8 months, pre-pregnancy maternal obesity, maternal smoking when child was age 12, maternal harmful alcohol consumption when child was age 12, maternal cannabis use when child was age 9 and maternal ever trouble with the law when child was age 12 [↑](#footnote-ref-2)
